# Supplementary material for: Dynamic recognition and mirage using neuro-metamaterials
Source: Nat Commun. 2022 May 16;13:2694. doi: 10.1038/s41467-022-30377-6 (PMC9110342; doi:10.1038/s41467-022-30377-6)
Supplement: Supplementary file 1 — Supplementary Information [file 41467_2022_30377_MOESM1_ESM.pdf]

Supplementary Information for

Dynamic recognition and mirage using neuro-metamaterials

Chao Qian[1,2,3,*], Zhedong Wang[1,2,3], Haoliang Qian[1,2,3], Tong Cai[1,2,3], Bin Zheng[1,2,3], Xiao Lin[1,2,3], Yichen Shen[4], Ido Kaminer[4], Erping Li[1,2,3,*], and Hongsheng Chen[1,2,3,*]

[1] ZJU-UIUC Institute, Interdisciplinary Center for Quantum Information, State Key Laboratory of Modern Optical Instrumentation, Zhejiang University, Hangzhou 310027, China.

[2] ZJU-Hangzhou Global Science and Technology Innovation Center, Key Lab. of Advanced Micro/Nano Electronic Devices & Smart Systems of Zhejiang, Zhejiang University, Hangzhou 310027, China.

[3] Jinhua Institute of Zhejiang University, Zhejiang University, Jinhua 321099, China.

[4] Lightelligence Inc., Boston, MA 02210, USA.

[5] Department of Electrical and Computer Engineering, Technion–Israel Institute of Technology, Haifa 32000, Israel.

**Corresponding author: chaoq@intl.zju.edu.cn (C. Qian); liep@zju.edu.cn (E. Li); hansomchen@zju.edu.cn (H. Chen)*

The PDF file includes:

- Supplementary Note 1: Mathematical model of neuro-metamaterials
- Supplementary Note 2: Rabbit modeling and data collection
- Supplementary Note 3: Training neuro-metamaterials
- Supplementary Note 4: Experimental setup and measurement
- Supplementary Note 5: Advantages over conventional transformation optics-based illusion

Supplementary Note 1: Mathematical model of neuro-metamaterials

Neuro-metamaterials can be designed in many forms, such as bulky, planar, layered, and layer-free metamaterials, assisted by diverse optimization algorithms, such as topology optimization, genetic algorithms, particle swarm algorithms, and deep learning. For simplicity, here we consider a diffractive neural network based on the conventional Huygens' principle. The underlying mechanism is that each point on a given wavefront can be considered as a center of a new originating disturbance, called the secondary source, that offers a new spherical wavelet [S1-S3]. In our design, the meta-atom with subwavelength scale can be treated similarly. As shown in Supplementary Fig. 1, the EM field of the meta-atom/neuron located at $\vec{r}^{l+1} = (x^{l+1}, y^{l+1}, z^{l+1})$ is contributed by all the neurons of the low-level layer. According to Rayleigh-Sommerfeld diffraction [28], the EM field can be expressed as

$$u(\vec{r}^{l+1}) = \iint_{-\infty}^{\infty} \frac{-1}{2\pi}\left(ik - \frac{1}{R}\right)\frac{y-y_s^l}{R}\frac{e^{ikR}}{R} \cdot u(\vec{r}_s^{\,l}) \cdot t(\vec{r}_s^{\,l}) dxdz \tag{S1}$$

where $\vec{r}_s^{\,l} = (x_s^l, y_s^l, z_s^l)$ refers to the location at the l-th layer, R refers to the distance between the source location $\vec{r}^{l+1}$ and observation point location $\vec{r}_s^{\,l}$, $t(\vec{r}_s^{\,l})$ represents the transmitted spectrum of the location $\vec{r}_s^{\,l}$, and k is the wavevector of light in free space. For simplicity, we rewrite Eq. (S1) in discrete form:

$$u_{p,q}^{l+1} = \sum_{m=1}^{M}\sum_{n=1}^{N} g_{m,n}^{p,q} \cdot u_{m,n}^{l} \cdot t_{m,n}^{l} \tag{S2}$$

where $g_{m,n}^{p,q} = \frac{-1}{2\pi}\left(ik - \frac{1}{R}\right)\frac{y-y_s^l}{R}\frac{e^{ikR}}{R}$. (m,n) represents a meta-atom located at the m-th column and n-th row. Similarly, (p,q) represents a meta-atom located at the p-th column and q-th row. The value of l ranges from 1 to L, which is the number of the metamaterials' layers. We further write Eq. (S2) in concise form:

$$u_k^{l+1} = \sum_{s=1}^{M\times N} g_s^k \cdot u_s^l \cdot t_s^l \tag{S3}$$

where $k = M \times (p-1) + q$ and $s = M \times (n-1) + m$. In matrix form, Eq. (S3) is expressed as

$$U^{l+1} = G^l \cdot (U^l \odot T^l) \tag{S4}$$

where $\odot$ refers to the Hadamard product, $U^l = \left(u_1^l, u_2^l, \cdots, u_{M\times N}^l\right)^T$, $T^l = \left(t_1^l, t_2^l, \cdots, t_{M\times N}^l\right)^T$, and

$$G^l = \begin{pmatrix} g_1^1 & \cdots & g_{M\times N}^1 \\ \vdots & & \vdots \\ g_1^{M\times N} & \cdots & g_{M\times N}^{M\times N} \end{pmatrix} \tag{S5}$$

Note that U^1 is directly the EM field induced by the object. Therefore, the intensity of the output plane is $(U^{L+1})^* \odot U^{L+1}$, in which $*$ represents the conjugate operation. The loss function is defined as

$$f = \frac{1}{M*N} \sum_{s=1}^{M\times N} |(u_s^{L+1})^* \odot u_s^{L+1} - (g_s^{L+1})^* \odot g_s^{L+1}| \tag{S6}$$

where g_s^{L+1} is the expected EM field (ground truth). Our goal is to minimize the loss function using a gradient-based method. The training process is shown in detail on the left-hand side of Supplementary Fig. 3.

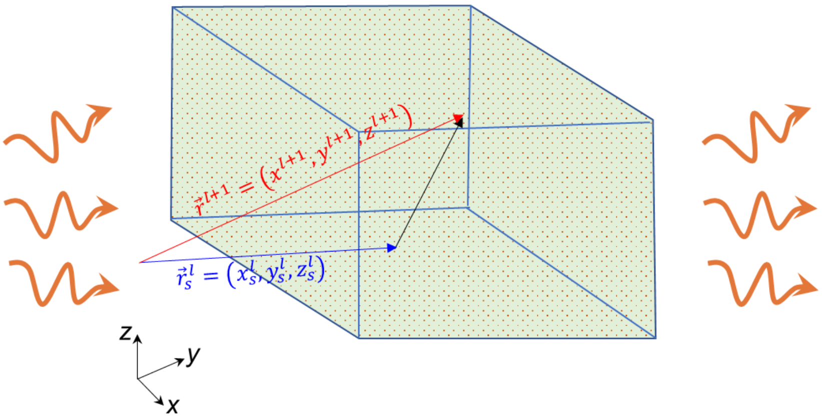

Supplementary Figure 1 | Mathematical model of the neuro-metamaterials. $\vec{r}_s^l = (x_s^l, y_s^l, z_s^l)$ represents the point (x_s^l, z_s^l) at the l-th layer. $\vec{r}^{l+1} = (x^{l+1}, y^{l+1}, z^{l+1})$ represents the point (x^{l+1}, z^{l+1}) at the $(l+1)$-th layer. The electromagnetic field of the meta-atom/neuron located at $(x^{l+1}, y^{l+1}, z^{l+1})$ is contributed by all the neurons of the low-level layer based on Huygens' principle.

Supplementary Note 2: Rabbit modeling and data collection

Data collection is a pivotal prerequisite for designing neuro-metamaterials. For this purpose, we conducted many numerical simulations, using the commercial software package CST Microwave Studio. The simulation setup is shown in Supplementary Fig. 2. When a plane wave is incident on a rabbit model, it will induce intricate scattered waves. The scatted waves are influenced by many factors, where we consider mainly the rabbit's posture and size, the distance between the rabbit and the layered metamaterials, and the rotation angle. All the situations are considered in the simulation, and thus, we create a dataset. The polarization state of the incident wave may be modified during scattering, but it does not appreciably influence the results, as our designed metamaterials are insensitive to EM polarization.

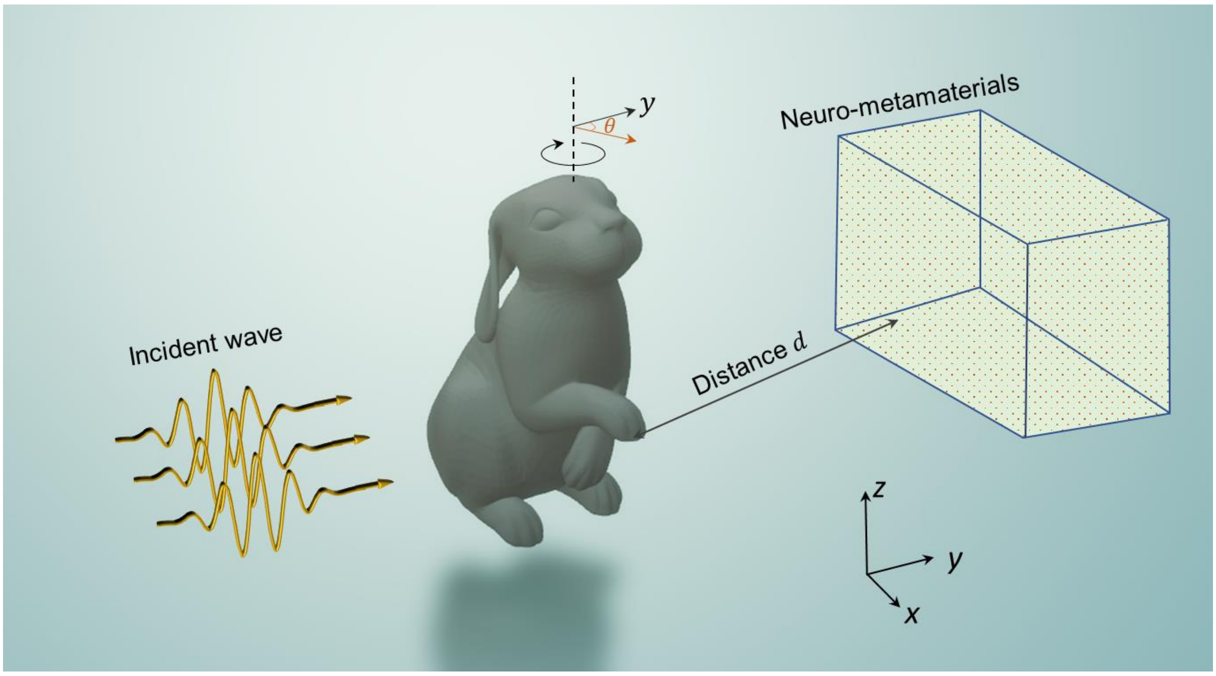

Supplementary Figure 2 | Simulation setup. A three-dimensional rabbit model is located in front of the neuro- metamaterials. The distance between the rabbit's center and the first metamaterial is denoted as d, which is tuned from 10 mm to 100 mm in steps of 10 mm. Initially, the rabbit's body is parallel to the neuro-metamaterials, and then, we gradually tune it; i.e., the rotation angle is varied from -30° to 30° in steps of 10°. In this way, we import these situations into the commercial software package CST Microwave Studio and continuously generate the data by using the MATLAB-CST co-simulation method. In addition, we consider different sizes of the rabbit and different postures.

Supplementary Note 3: Training neuro-metamaterials

The flow chart of the neuro-metamaterials training process is shown in Supplementary Fig. 3a. We input the scattered field into a designed diffractive neural network architecture and calculated the loss of the neural network to update the transmitted spectra of the metamaterials iteratively, until the neural network converged. To facilitate the design and fabrication of the metamaterial used in the experiment, we consider only the transmitted phase modulation with the transmitted amplitude being unity. The neuro-metamaterials are trained using Python version 3.5.0. and TensorFlow framework version 1.10.0 (Google Inc.) on a server (GeForce GTX TITAN X GPU and Intel(R) Xeon(R) CPU X5570 @2.93GHz with 48GB RAM, running a Linux operating system). It takes dozens of minutes for our neuro-metamaterials to converge. The ultimate phase profiles are shown in Supplementary Fig. 3b. The design principle and pre-trained phase masks can be scaled into other single frequency. For different frequencies, we should design specific metamaterials due to material dispersions to match the pre-trained phase masks, such as TiO_2 metasurfaces in visible [35].

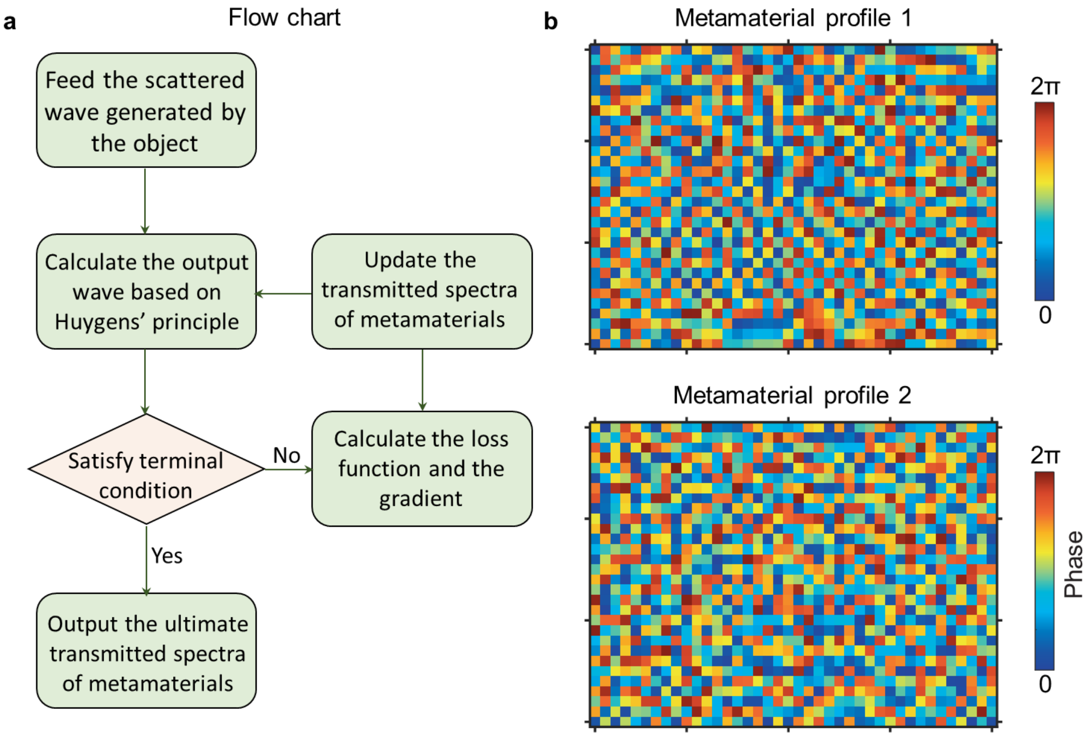

Supplementary Figure 3 | Training results for the neuro-metamaterials. a, Flow chart of the training process. We fed the scattered waves generated by the object into a diffractive neural network and calculated the loss function and the gradient to update the transmitted spectra of

metamaterials repeatedly until the network converged. **b**, The ultimate transmitted phase distributions of the neuro- metamaterials.

Supplementary Note 4: Experimental setup and measurement

Our experiment was conducted in a microwave anechoic chamber to avoid environmental interference, and its configuration included mainly a high-directivity lens antenna, vector network analyzer, and back-end detection system, as shown in Supplementary Fig. 4a. The high-directivity lens antenna is perpendicular to the neuro-metamaterials, which are connected to a vector network analyzer to excite the EM waves. When a rabbit plays freely in front of the neuro-metamaterials, we perceive the signal in real time by means of the back-end detection system (Supplementary Fig. 4b). The results in Fig. 3 of the main text were obtained in this manner.

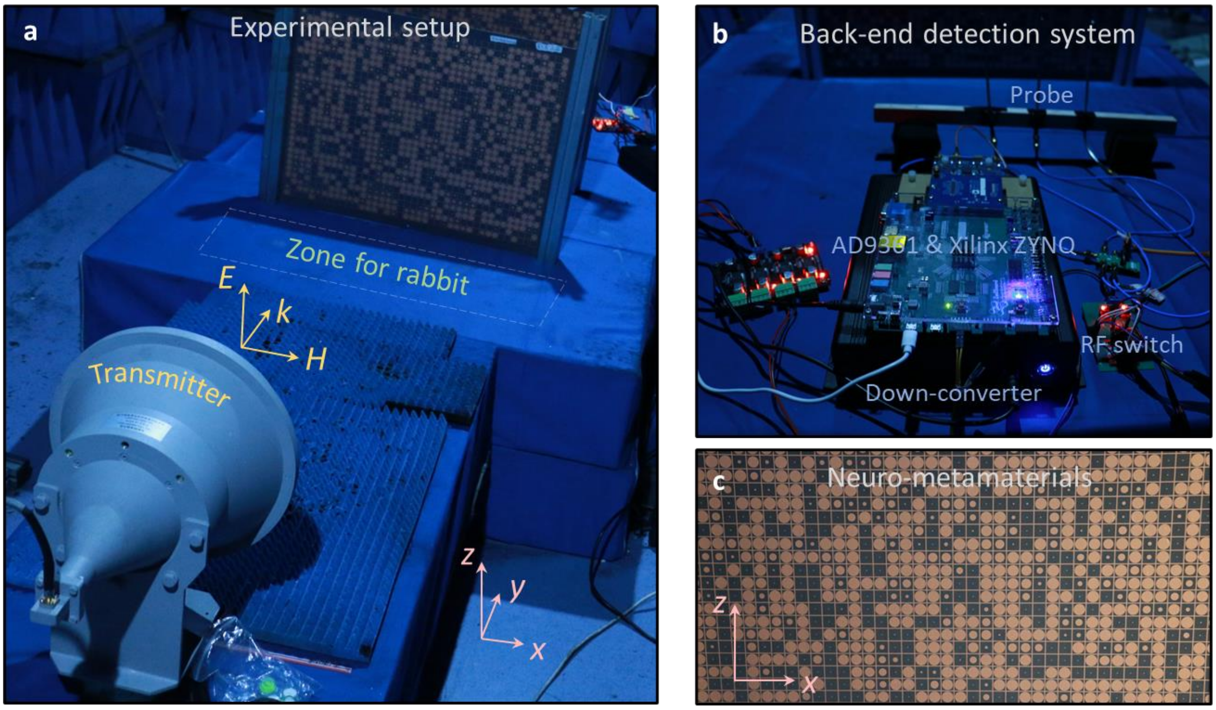

Supplementary Figure 4 | Experimental configuration. a, A high-directivity lens antenna excites the TE plane wave toward the neuro-metamaterials. Behind the neuro-metamaterials, there are three home-made single-pixel monopole probes used to detect the amplitude of the EM waves. **b**, Back-end detection system. The received signal (8.6 GHz) passes through a broadband amplifier and mixer (6.0 GHz). Then, we employed an AD9361, Xilinx ZYNQ for data processing accelerated by a field-programmable gate array (FPGA). **c**. Zoomed-in view of the experimental metamaterials, fabricated using a standard printed circuit-board method.

Supplementary Note 5: Advantages over conventional transformation optics-based illusion

In 2009, optical illusion using transformation optics and complementary media was first theoretically proposed [23]. In addition, there exist many studies based on similar mechanisms [S4], such as a superscatterer [S5,S6] and a remote cloak [S7,S8]. In general, these studies have a common feature: the composite materials used were typically negative, inhomogeneous, and anisotropic. Hence, experimental realization of the theory is extremely challenging. In addition, for conventional optical illusion, the mechanisms always operate in a static state, and any movement of the object may significantly degrade the performance of even a well-designed mechanism. These challenges greatly hinder their further development.

In stark contrast, our newly proposed approach can efficiently bypass these difficulties and open a new avenue. First, we need only to modulate the transmitted spectra of the metamaterials, which can be easily accessed by current research methods. Second, through a moderate optimization, the layered metamaterials can successfully create an illusion in the case of dynamic objects. These two overwhelming advantages can to a great extent overcome the long-standing obstacles to the realization of optical illusion.

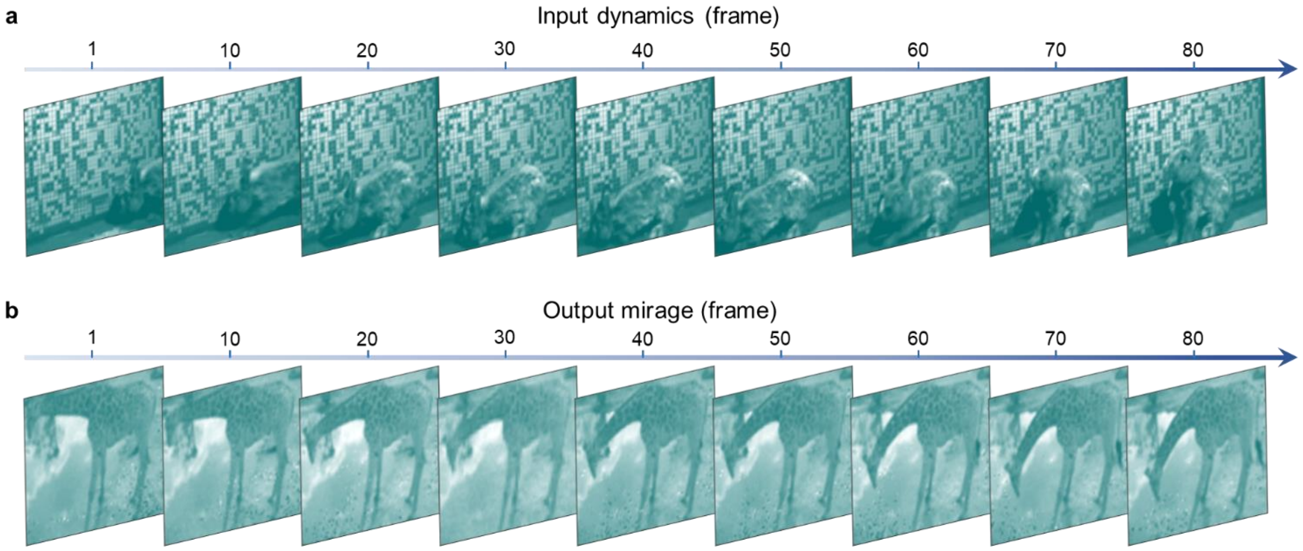

Supplementary Figure 5 | Another dynamic illusion example that incorporates more images as an optimization task. a, Snapshots of a moving rabbit. **b**, Output mirage.

We would like to elucidate that neuro-metamaterials can be applied for inference-based and optimization-based tasks, both of which are useful for different application demands. In our work, we demonstrate the two capabilities via posture recognition and optical illusion experiments, respectively. Optical illusion in the main text (Fig. 4) is treated as an optimization task. Although the

number of input images is small in the proof-of-the concept experiment, we can still observe the continuous movement of rabbit and giraffe; this presented dynamic illusion actually outperforms most of previous illusions working for a single input. Towards real-world applications, one can incorporate more desired images with a more sophisticated network, as shown in Supplementary Figs. 5 and 6.

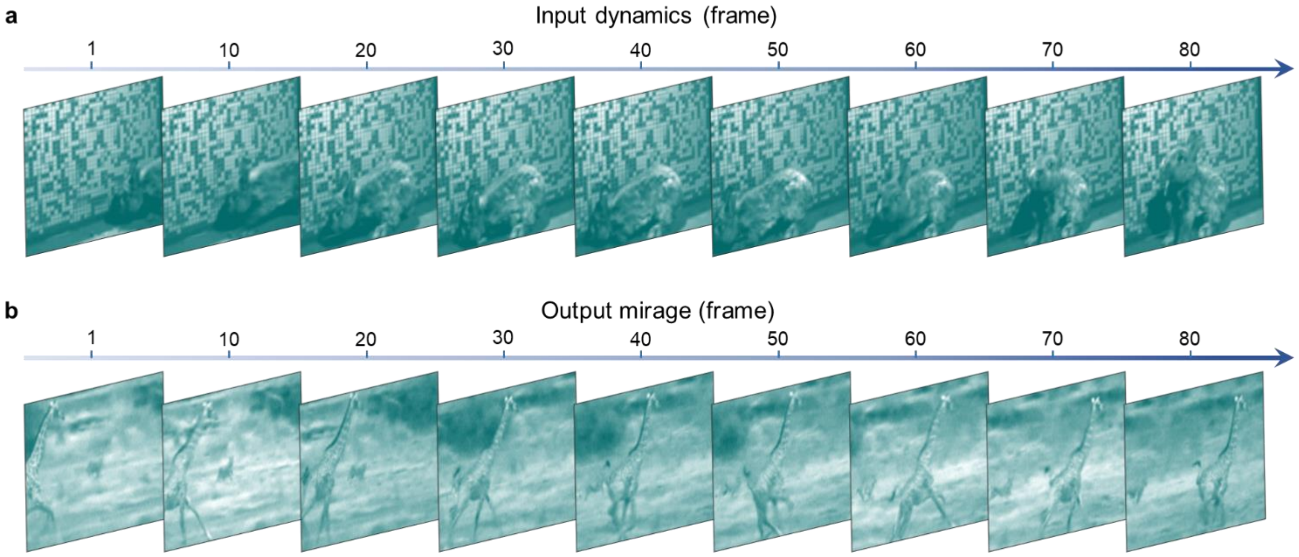

Supplementary Figure 6 | Dynamic illusion example of a running giraffe. a, Snapshots of a moving rabbit. **b**, Output mirage.

On the other hand, we can also deal with optical illusion as an inference (machine learning) task using neuro-metamaterials. Such generalization capability actually has been well verified in our first posture recognition experiment. To benchmark this again, we carry out another illusion experiment to transform rabbit (with different postures of walking, sitting, and standing) into a drinking giraffe with different postures based on the dataset in the first posture recognition experiment. After adequate training, neuro-metamaterials can work for untrained images to produce a dynamic illusion (Supplementary Fig. 7). Even when the rabbit's head raises higher, the output giraffe's head goes down.

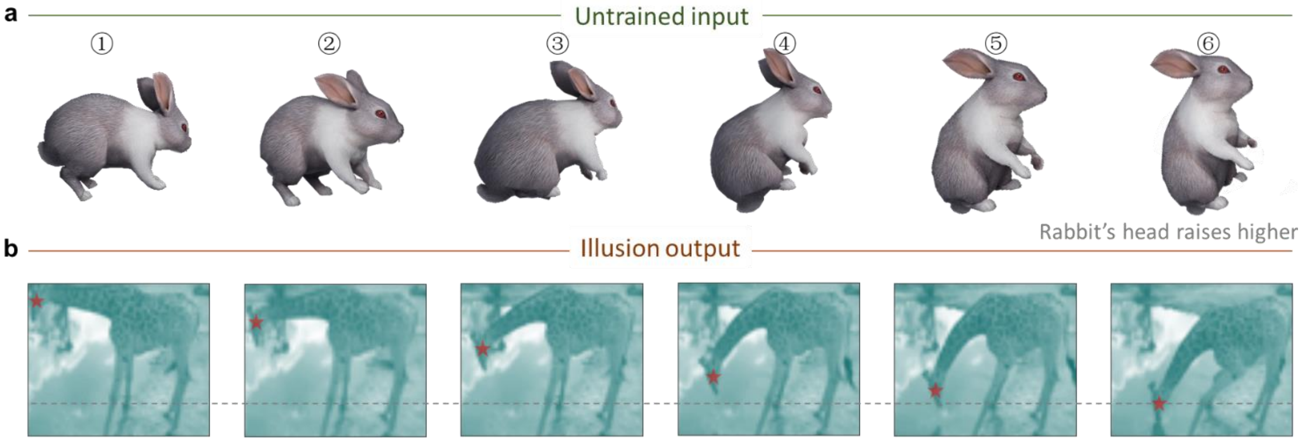

Supplementary Figure 7 | Illusion results for untrained input as an inference task. a, Different rabbit postures. **b**, Output mirage. In this experiment, we treat dynamic illusion as an inference task, aiming to transform rabbit (with different postures of walking, sitting, and standing) into a drinking giraffe. The five-pointed stars represent the locations of the giraffe's head and the dotted line is added as a reference. When the rabbit's head raised higher, the giraffe's head goes down gradually. For ⑥, the rabbit's head is the highest and does not appear among the training samples.

Supplementary References

[S1] Born, M. and Wolf, E. *Principles of Optics: Electromagnetic Theory of Propagation, Interference and Diffraction of Light* 7th edn (Cambridge Univ. Press, 1999).
[S2] Rahman, M. S. S., Li, J. Mengu, D., Rivenson, T., & Ozcan, A. Ensemble learning of diffractive optical networks. *Light Sci. Appl.* **10**, 14 (2021).
[S3] Yan, T. *et al.* Fourier-space diffractive deep neural network. *Phys. Rev. Lett.* **123**, 023901 (2019).
[S4] Wee, W. H. and Pendry, J. B. Shrinking optical devices. *New J. Phys.* **11**, 073033 (2009).
[S5] Luo, X. *et al*. Conceal an entrance by means of superscatterer. *Appl. Phys. Lett.* **94**, 223513(2009).
[S6] Lai, Y. *et al.* Complementary media invisibility cloak that cloaks objects at a distance outside the cloaking shell. *Phys. Rev. Lett.* **102**, 093901 (2009).
[S7] Qian, C. *et al.* Observing the transient buildup of a superscatterer in the time domain. *Opt. Express* **25**, 4967-4974 (2017).
[S8] Zheng, B. *et al.* Concealing arbitrary objects remotely with multi-folded transformation optics. *Light Sci. Appl.* **5**, e16177 (2017).
