# Supplementary material for: Dynamic recognition and mirage using neuro-metamaterials
Source: Nat Commun. 2022 May 16;13:2694. doi: 10.1038/s41467-022-30377-6 (PMC9110342; doi:10.1038/s41467-022-30377-6)
Supplement: Supplementary file 3 — Description of Additional Supplementary Files [file 41467_2022_30377_MOESM3_ESM.pdf]

Description of Additional Supplementary Files

File name: Supplementary Movie 1

Description: A large grey rabbit plays freely in front of the neurometamaterials; the time-varying signals perceived by three single-pixel detectors are attached.

File name: Supplementary Movie 2

Description: A small black-white five months-old rabbit plays freely in front of the neuro-metamaterials; the time-varying signals perceived by three single-pixel detectors are attached.

Description: Supplementary Movie 3

Description: Dynamic optical mirage, where the input is image frames extracted from the Supplementary Movie 1 and the output is an online giraffe video. In Supplementary Movie 3, we provided the raw image frames and the sampled images that are used for training neuro-metamaterials. Notice that, to facilitate watching, the second half of Supplementary Movie 3 was made by reversing its first half.
