# Supplementary figures and images for: Dynamic recognition and mirage using neuro-metamaterials
Source: Nat Commun. 2022 May 16;13:2694. doi: 10.1038/s41467-022-30377-6 (PMC9110342; doi:10.1038/s41467-022-30377-6)

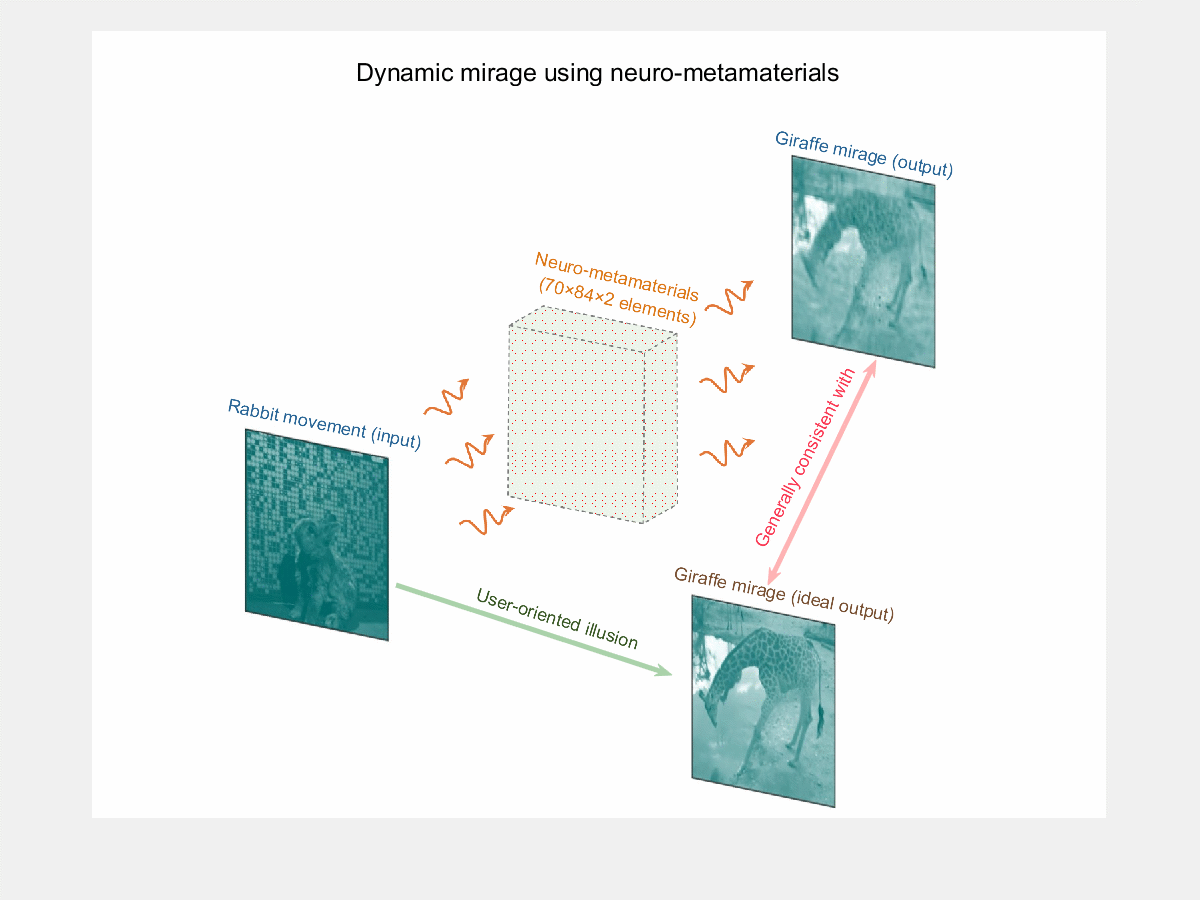

Supplement: Supplementary file 6 — Supplementary Movie 3 [file 41467_2022_30377_MOESM6_ESM.gif]
